# Supplementary material for: Saccharomyces boulardii (CNCM I-745) improves intestinal damage in sepsis by remodeling intestinal flora structure
Source: Front Cell Infect Microbiol. 2025 Jul 31;15:1602792. doi: 10.3389/fcimb.2025.1602792 (PMC12350329; doi:10.3389/fcimb.2025.1602792)
Supplement: Supplementary file 1 [file DataSheet1.zip › summary/summary/7_Differential_analysis/1_diff_abundance/7_Species/Group.BW4_vs_BW0/Group.BW4_vs_BW0_diff_barplot.pdf]

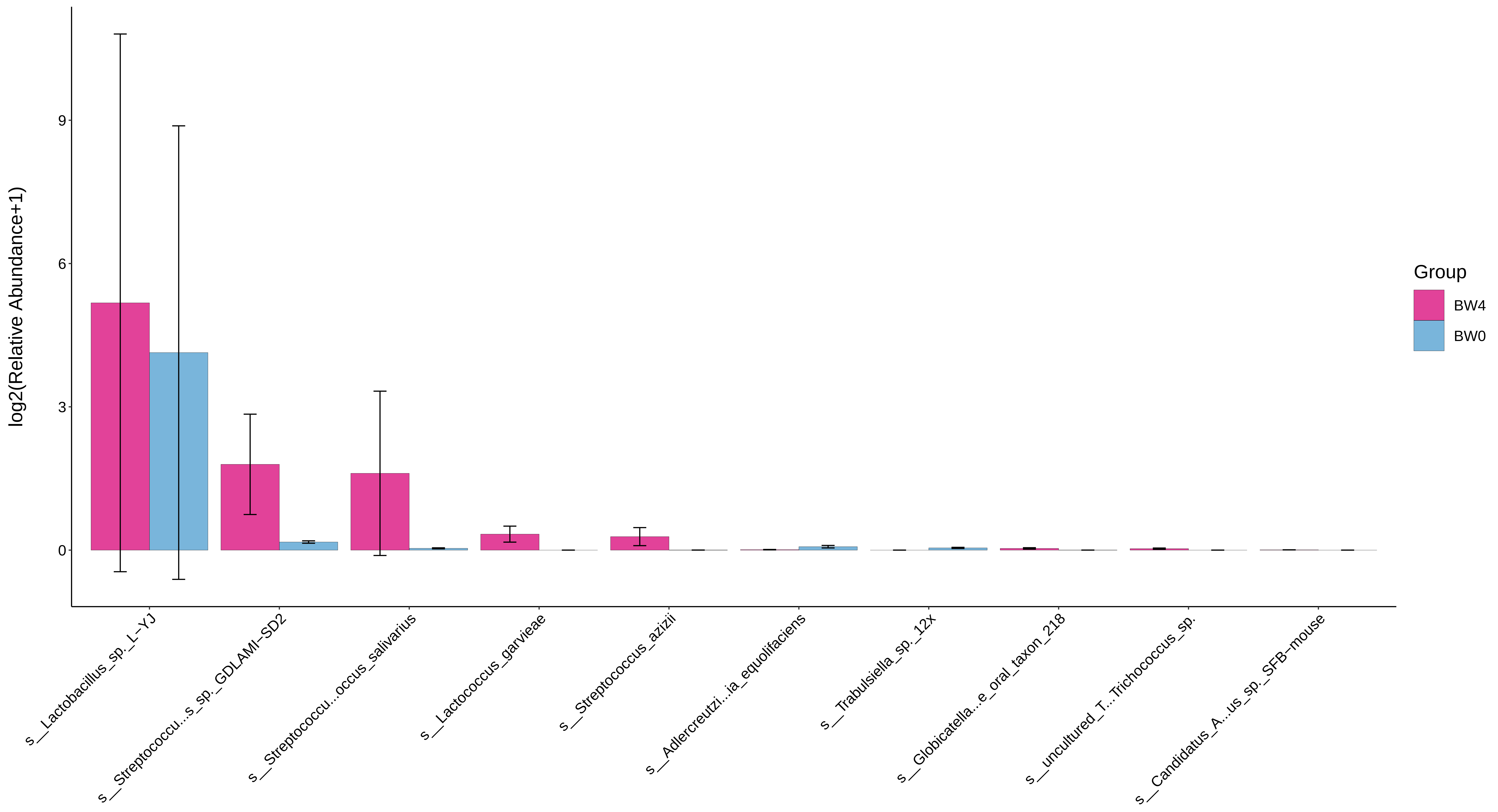

log2(Relative Abundance+1)
0
3
6
9
Group
BW4
BW0
s__Lactobacillus_sp._L−YJ
s__Streptococcu...s_sp._GDLAMI−SD2
s__Streptococcu...occus_salivarius
s__Lactococcus_garvieae
s__Streptococcus_azizii
s__Adlercreutzi...ia_equolifaciens
s__Trabulsiella_sp._12x
s__Globicatella...e_oral_taxon_218
s__uncultured_T...Trichococcus_sp.
s__Candidatus_A...us_sp._SFB−mouse
